# Structural and mechanistic insights into the VP14460-VP14465 effector-immunity module of the *Vibrio parahaemolyticus* type VI secretion system

**DOI:** 10.1016/j.jbc.2026.113257

**Published:** 2026-06-15

**Authors:** Yuyan Zheng, Chenhua Zheng, Zhouyang Ye, Lirui Huang, Xuezhongqing Lin, Binjie Wu, Zihang Pan, Rui Qiu, Jinyuan Cai, Linchen Xu, Zilin Deng, Ruxin Xu, Xi Xie, Lihua Xie, Fen Hu

**Affiliations:** 1 Key Laboratory of Ministry of Education for Gastrointestinal Cancer, School of Basic Medical Sciences, Fujian Medical University, Fuzhou, China; 2 State Key Laboratory of Membrane Biology, MOE Key Laboratory of Bioinformatics, Tsinghua-Peking Center for Life Sciences, School of Life Sciences, Tsinghua University, Beijing, China; 3 State Key Laboratory of Drug Research, Shanghai Institute of Materia Medica, Chinese Academy of Sciences, Shanghai, China

**Keywords:** crystal structure, effector-immunity, VP14460-VP14465, T6SS, toxin

## Abstract

*Vibrio parahaemolyticus*, a halophilic pathogen, contaminates seafood and high-salt foods, posing significant health risks such as gastroenteritis and fatalities. With increasing seafood consumption, developing effective control strategies is imperative. The type VI secretion system, a common molecular weapon in *Vibrio* that mediates cross-domain interactions, is a contractile nanomachine that deploys antibacterial effectors, each paired with a cognate immunity protein to form effector-immunity (E-I) modules. Here, we present the first structural and functional characterization of this complex. The crystal structure of the VP14460-VP14465 complex unveils the molecular basis for specific E-I recognition, defining a set of critical interfacial residues. Structure-guided mutagenesis confirmed that these residues are essential for complex integrity, their disruption liberates the VP14465 toxin, unleashing potent bactericidal activity. Furthermore, we identified the active sites responsible for the DNase activity of VP14465. Notably, while VP14460 and VP14465 form a stable monomeric complex in the bound state, each isolated component exhibits distinct oligomeric behavior *in vitro*. This suggests a dynamic assembly-disassembly mechanism that may regulate effector delivery and toxin activation during interbacterial competition. Collectively, our findings provide mechanistic insights into type VI secretion system E-I module architecture and function, and establish a structural framework for the development of novel antibacterial therapeutics targeting DNase family effectors.

Foodborne pathogens pose a major threat to global food safety and public health.The World Health Organization reports that pathogenic microorganisms cause 70% of annual foodborne disease outbreaks and 1.8 million deaths (1). Antibiotic misuse has exacerbated bacterial resistance, making multidrug-resistant strains a growing concern (2).*Vibrio parahaemolyticus*, a halophilic bacterium prevalent in marine ecosystems, contaminates seafood and high-salt foods, causing severe gastroenteritis, dehydration, and fatalities (3, 4). Widely distributed in coastal waters, *V. parahaemolyticus* is a major foodborne pathogen responsible for seafood-associated gastroenteritis in humans. It serves as the primary causative agent of acute hepatopancreatic necrosis disease in shrimp (5). Alarmingly, strains from seafood and clinical sources show high antimicrobial resistance, fueled by long-term antibiotic use (4, 6). As seafood consumption rises, developing effective, low-resistance control strategies is crucial to mitigate *V. parahaemolyticus* contamination.

Evidence indicates that bacterial survival within polymicrobial communities relies on the synthesis of interbacterial antagonistic factors. Moreover, the natural competency of *Vibrio* species, coupled with horizontal gene transfer, drives the emergence of new pathogenic strains by facilitating the acquisition of novel virulence traits (7, 8). In marine ecosystems, *Vibrio* spp. actively compete with adjacent bacterial communities for limited resources while establishing complex ecological relationships with protists, which may act either as predators providing selective pressure or as transient hosts offering replicative niches (9, 10, 11).

The type VI secretion system (T6SS), a molecular weapon widespread among *Vibrio* species, acts as a key mediator of cross-domain interactions. It facilitates both interbacterial competition and engagement with eukaryotic host (12, 13). T6SS components also serve as antigens used in vaccines development for clinical trials, and as future vaccine candidates (14). T6SS has a modular structurewith a membrane complex (TssJ/L/M), a baseplate (TssE/F/G/K), and an Hcp protein inner tube surrounded by a TssB/TssC contractile sheath (15, 16). When assembled, sheath contraction propels the Hcp tube and its tip complex-comprised of cone-shaped VgrG and PAAR domain protein (with Pro-Ala-Ala-Arg repeats), into target cells. This enables delivery of effector proteins (17, 18). The contractile nanomachine delivers a variety of antibacterial effectors (*e.g.*, phospholipases, peptidoglycan hydrolases, nucleases, membrane pore-forming proteins) that target four core cellular components: cell wall integrity (glycoside hydrolases), membrane stability (lipases), genetic material (nucleases), and energy metabolism (NAD+/NADH-targeting enzymes) (19, 20, 21). Each offensive effector is genetically paired with a cognate immunity protein in adjacent gene clusters, forming effector-immunity (E-I) modules -a key adaptation, as T6SS lacks intrinsic self/nonself discrimination (22, 23, 24). These E-I modules both protect producer cells from self-intoxication and defend against competing bacteria. This is evidenced by well-characterized systems such as TseH-TsiH in *Vibrio cholerae*, TplE-TplEi and Tse6-Tsi6 in *Pseudomonas aeruginosa*, and polymorphic nuclease effectors (PoNe) with their cognate immunity proteins (polymorphic nuclease immunity) in *V. parahaemolyticus* (25, 26, 27).

*V. parahaemolyticus* encodes two distinct T6SSs. T6SS1 mediates antibacterial activity during interbacterial competition by delivering strain-specific effector repertoires (28). Several MIX (Marker for type sIX effectors)-containing proteins have been identified as T6SS1 effectors, and studies confirm that VP1388-VP1389, VPA1263-Vti2, and VP1415-VP1416 function as E-I pairs (29, 30). In addition, RhsP, which contains a PAAR motif, forms an E-I pair with RhsPi and contributes to the policing of social cheaters in the *V. parahaemolyticus* community (31). Notably, Tme-family effectors localize to the periplasm and intoxicate target cells by disrupting membrane integrity (32). In contrast, T6SS2 is conserved across all *V. parahaemolyticus* isolates and has recently been implicated in interbacterial competition. Its secreted effectors include the “core” components T2RhsNuc and T2LipB, as well as accessory effectors such as T2LipA, T2Hydro, T2Tme, and T2Unkwn (5). Furthermore, two duplicated phospholipase effectors of T6SS2, TleA and TleB, have been demonstrated to mediate interspecies competition (33). Despite the growing number of identified T6SS E-I modules, the structural mechanisms by which immunity proteins neutralize cognate toxins or how these toxins exert their toxicity to eliminate competitor cells remain poorly understood.

Recently, a new T6SS1 E-I module, VP14460-VP14465, was identified (34). The effector protein VP14465 (PoNe) belongs to a distinct superfamily of PD-(D/E)xK phosphodiesterases and is linked to type V, VI, and VII secretion systems. This novel toxin family’s activity is neutralized by its immunity protein VP14460 (polymorphic nuclease immunity), which contains DUF1910 and DUF1911 domains. This E-I module is widely found in the secretion systems of various antibacterial toxins in both Gram-negative and Gram-positive bacteria. Here, we report its structural and biochemical characterization. Our studies reveal the key intermolecular interactions between VP14460 and VP14465. Mutagenesis experiments confirm these interactions are crucial for their association. This work provides a structural framework for understanding the molecular mechanisms of pathogenicity in human pathogens and supports development of T6SS-based vaccines against *V*. *parahaemolyticus*.

## Results

### Neutralization of VP14465 toxicity by VP14460

**Figure 1:**
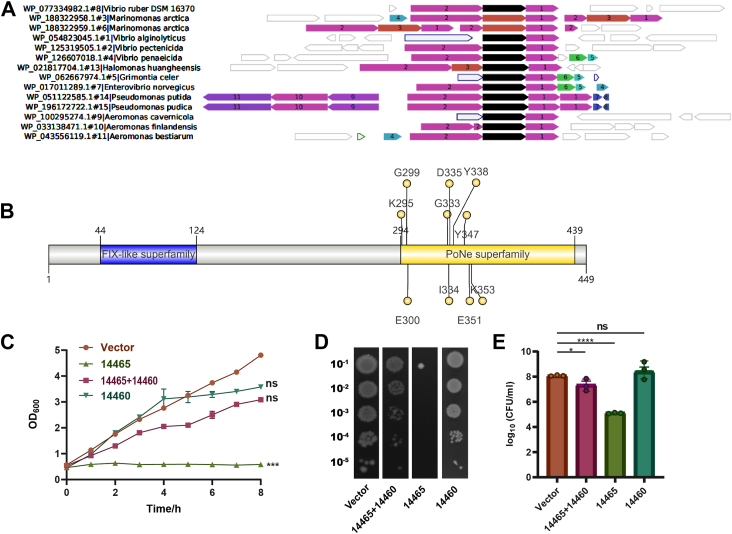
**Identification of VPS4460-VPS4465 as an E-I system.***A*, comparative genomic analysis of *vp14465* locus using WebFlaGs. The input gene (*vp14465*, *black-shaded regio*n) and its neighboring genes are shown. Gene 1 (*vp14460*, or encodes a DUF1911 domain-containing protein) is directly adjacent to *vp14465*, suggesting they belong to the same operon, gene 2 encodes a T6SS tip protein VgrG, gene 3 encodes a hypothetical protein, gene 4 encodes T6SS effector Hcp1, gene 5 encodes T6SS PAAR protein, gene 6 encodes a hypothetical protein, gene 7 encodes PAAR domain-containing protein, gene 8 encodes a DUF6026 family protein, gene 9 encodes glycogen synthase GlgA, gene 10 encodes malto-oligosyltrehalose trehalohydrolase, gene 11 encodes 4-alpha-glucanotransferase. *B*, illustrator for biological sequence of VP14465 by IBS 2.0 software (https://ibs.renlab.org). The scale marked on the map indicates relative residues positions. *C*, the effects of VP14465 production on the culture density of *Escherichia coli* strains with or without VP14460. Culture densities were enumerated by OD_600_ at subsequent time points. *D* and *E*, the survival of *E. coli* cells was examined by serial dilution by dilution spot tests. All cultures were grown in LB medium and induced with IPTG at time zero with an OD_600_ value of 0.5. Record the OD_600_ value of the bacterial suspension once per hour. After further incubation, cells were plated to enumerate CFU. Each point represents the average of triplicate values, error bars represent standard deviation. One-way ANOVA was employed to compare the growth curves and CFU assay results with the vector control group. Statistical significance is indicated as follows: ns, not significant, ∗*p* < 0.05, ∗∗*p* < 0.01, ∗∗∗*p* < 0.001, ∗∗∗∗*p* < 0.0001. CFU, colony-forming unit.

To investigate the evolutionary conservation and potential functional relationship between *vp14465* and its neighboring genes, we performed comparative genomic analysis using WebFlaGs (35). The results showed that *vp14460* is evolutionarily conserved and located in close proximity to *vp14465* across various species (Fig. 1*A*), implying a potential functional association between these two genes. To determinate whether they belong to the E-I module, we attempted to separately purify the full-length soluble proteins of VP14460 and VP14465. VP14460 was successfully purified, whereas repeated attempts to express VP14465 were unsuccessful. Sequence analysis revealed that VP14465 shares no homology with any known toxin domains. NCBI gene annotation indicated that VP14465 contains an N-terminal fixed sample superfamily and a C-terminal PoNe superfamily domain (residues 294–439) (Fig. 1*B*) (36). According to this domain organization, we expressed the PoNe superfamily region (designated VP14465C, the C-terminal region of full-length VP14465, which was used for structural and biochemical analyses.) in a prokaryotic system. Functional verification confirmed that this domain is both necessary and sufficient to exert toxic effects, as reflected by inhibited growth of *Escherichia coli* (Fig. 1*C*). In contrast, simultaneous co-expression of VP14460 and VP14465C restored normal bacterial growth (Fig. 1*C*), demonstrating that they act as an effector and its cognate immunity, respectively. This conclusion was further validated *via* spot dilution assays evaluating the survival capacity of *E. coli* cells in serial dilution experiments (Fig. 1, *D* and *E*).

### The Overall Dimeric Architecture of VP14460

**Figure 2:**
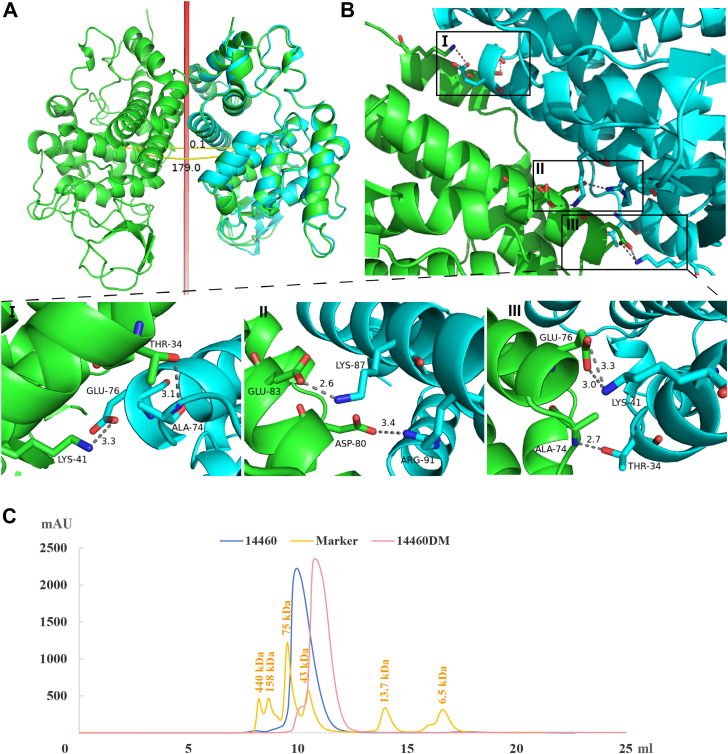
**The Overall Dimeric Architecture of VP14460.***A*, dimeric structure of VP14460. Chain A is colored in *green*, Chain *B* is colored in *cyan*, and the *red stick* denotes the rotation axis. *B*, intermolecular interactions of VP14460 dimer. The residues involved are shown as *sticks*. *C*, size exclusion chromatography (Superdex 75) elution profile/peaks of proteins monitored by absorbance at 280 nm.

The crystal structure of VP14460 was determined in space group *C*121 with resolution at 2.32 Å. The two asymmetric units of VP14460 formed a dimer with rotation axis direction cosines of (−0.18, −0.98, −0.09), a rotation angle of 178.99°, a translation of 0.05 Å along the axis, and a mass center distance of 33.56 Å (Fig. 2*A*). We carefully examined the topology of the VP14460 dimer and found that it was stabilized by seven pairs of intermolecular H-bonds distributed across three distinct regions (Fig. 2*B*), Region I: The hydroxyl group of the T34-A′ side chain establishes a hydrogen bond with the main-chain amide of A74-B’. Additionally, the amino group of the K41-A′ side chain forms a hydrogen bond with the carboxyl group of the E76-B′ side chain. Region II: Structural analysis revealed that the hydroxyl groups of D80 and E83 (chain A) form hydrogen bonds with the side-chain amides of R91 and K87 (chain B) at distances of 3.4 Å and 2.6 Å, respectively, suggesting electrostatic stabilization between these oppositely charged residues. Region III: The interfacial residues between the two molecules closely resemble those in region I but are contributed by the opposing chains. Upon simultaneous mutation of the involved amino acids (T34, K41, E76, D80, E83, K87, R91) to alanine (14460DM), size exclusion chromatography analysis revealed that the purified protein existed as a monomer (Fig. 2*C*, pink).

### VP14460 contains a DUF1911 domain

**Figure 3:**
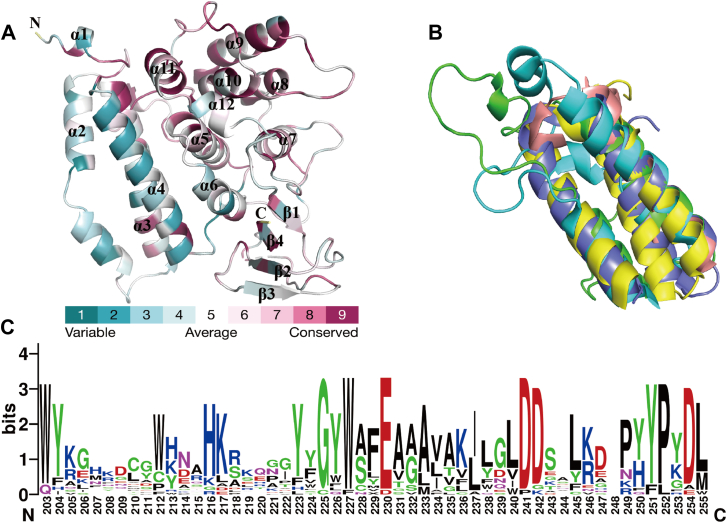
**Crystal structure of VP14460.***A*, cartoon representation and ConSurf analysis of the VP14460 structure using nine-color conservation scores. *B*, superposition of VP14460 (residues 194–201, *green*) with the highest-scoring DUF1911 structures, Protein Data Bank codes 2FEF, 6W2W, 7T1S, and 7NJ1, depicted in *cyan, wheat, blue*, and *yello*w, respectively. *C*, multiple sequence alignment of the homologs in NCBI by WebLogo.

Each monomeric VP14460 predominantly adopts a modified α/β-hydrolase fold (Fig. 3*A*). The N-terminus consist of twelve α-helices, its center region is formed by α3, α4, α5, α6 and α11, among which the adjacent helices α4 and α5 cross each other at an angle of approximately 60°, while the C-terminus forms a β-barrel composed of a four-stranded β-sheet. Residues 311 to 315 show prominent structural disorder, as indicated by the lack of interpretable electron density in the crystallographic data. Furthermore, evolutionary conservation analysis of homologous sequences performed using ConSurf reveals that multiple residues of VP14460 are highly conserved, especially within functional and structural motifs spanning residues 176 to 260, which maps to α-helices α8-α11 (Figs. 3*A* and S1). Notably, this region corresponds to the NCBI-annotated DUF1911 domain, which is widely present in bacterial genera including *Vibrio*, *Shewanella*, *Aeromonas*, and *Marinomonas* (Fig. S2). Structural homology search using DALI (37) further demonstrated that the DUF1911 domain possesses conserved modular architecture and secondary structure profile, with the top three homologous structures yielding considerable Z-scores of 5.1, 4.2, and 4.1. Comparative structural analysis shows high structural similarity between VP14460 and the highest-scoring DUF1911 homologs, particularly in the α8-α10 helices (Fig. 3*B*). Additionally, sequence alignment confirmed strong amino acid conservation in this region (Fig. 3*C*). The DUF1911 domain serves as a cognate immunity protein for the PoNe, which neutralizes its DNase toxicity *via* direct binding to prevent self-intoxication in bacterial T6SS (34, 38, 39).

### Overall structure of VP14460 in complex with the C-terminal domain of VP14465

**Figure 4:**
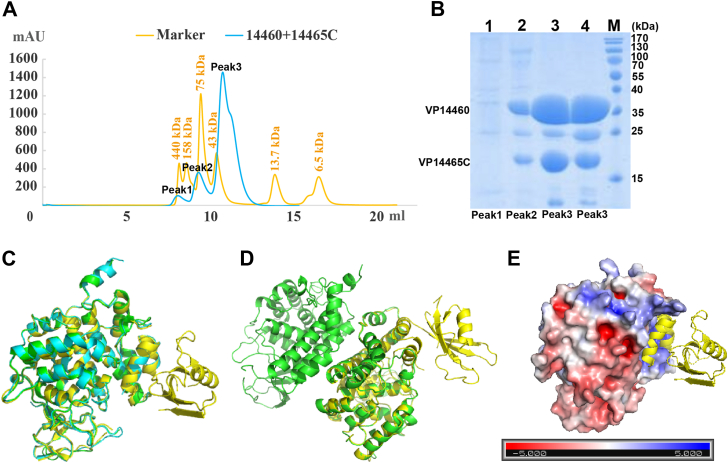
**Structure of the VP14460-VP14465 complex.***A*, size exclusion chromatography (Superdex 75) elution profile/peaks of proteins monitored by absorbance at 280 nm. *B*, SDS-PAGE of elution fractions gel filtration chromatography after affinity pull-down assays by His-VP14460 and GST-VP14465. M: Marker; lane 1 to 4: elution fractions of peaks. *C*, Structure superimposition of VP14460 (*green*), VP14460-VP14465 (*yellow*) and VP14460-VP14465pep (*cyan*). *D*, structural superposition of VP14460 (*green*) and the VP14460-VP14465 complex (*yellow*). *E*, the electrostatic surface potential of VP14460 in the VP14460-VP14465 structure (*red, blue* and *white* indicate negative, positive and neutral potentials, respectively).

To investigate the interaction between VP14460 and the C-terminal domain of VP14465, we co-expressed these proteins with distinct purification tags and isolated the protein complex using affinity pull-down assays. Subsequent size-exclusion chromatography analysis revealed that the VP14460-VP14465C complex elutes as a monomeric species. This conclusion was supported by its elution profile: the complex exhibited a peak elution volume of 10.88 ml (Fig. 4*A*), which was later than that of the purified VP14460 dimer (9.95 ml) but consistent with that of the VP14460DM monomer (10.80 ml). SDS-PAGE analysis of the main peak fraction (peak 3, Fig. 4*B*) confirmed the presence of both VP14460 and VP14465. This observation suggests that VP14465 binding may disrupt the native dimeric state of VP14460.

The crystal structure of the VP14460-VP14465C complex was determined in space group *P*12_1_1. Structural comparison between the VP14460-VP14465C complex and the VP14460 monomer revealed that the VP14460 moiety adopts a nearly identical fold, with a RMSD of 0.35 Å for Cα atoms (Fig. 4*C*). Notably, the dimerization interface of VP14460 differs from its binding interface with VP14465, with the two interaction sites oriented in distinct directions (Fig. 4*D*), indicating that the different domains of VP14460 are responsible for distinct biological functions. Electrostatic surface potential analysis further demonstrated that the VP14460 binding pocket exhibits a strongly acidic character (Fig. 4*E*), with the surrounding positively charged residues highlighted in Fig. S3.

### Molecular basis for recognition of VP14465 peptide bound to VP14460

**Figure 5:**
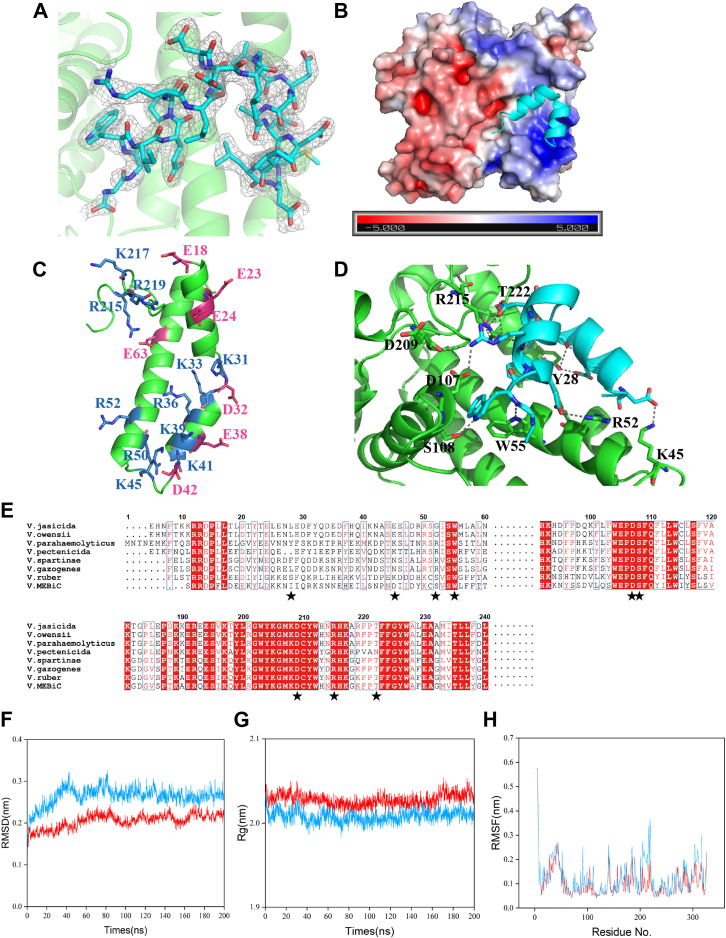
**Detailed interactions between VP14460 and VP14465 peptide.***A*, electron density map of the bound VP14465peptide. The 2Fo-Fc electron density map for VP14465pep (residues 394–416) is shown as a *gray* mesh contoured at 1.0 σ. The peptide is shown as *cyan sticks*. The map was calculated using PHENIX and visualized in PyMOL. *B*, electrostatic surface potential of VP14460 in the VP14460-VP14465pep complex. Negative, positive, and neutral potentials are colored *red*, *blue*, and *white*, respectively. The VP14465 peptide is depicted as a *cyan cartoon*. *C*, the charged residues at the VP14460 binding pocket, these residues are highlighted as *sticks*. The electrostatic potential was calculated using APBS and visualized in PyMOL. *D* detailed interactions between VP14460 and VP14465 peptide, and the residues involved are shown with a stick model, in which the backbones are colored *white* and *green*, respectively, and the nitrogen and oxygen atoms are colored *blue* and *red*, respectively. The VP14460 and VP14465 peptide are depicted as *green* and *cyan cartoo*n, respectively. *E*, sequence alignment of VP14460 from *V. parahaemolyticus*, *V. jasicida*, *V. owensii*, *V. pectenicida*, *V. spartinae*, *V. qazogenes* and *V. MEBiC*. The strands are numbered according to VP14460 in *V. parahaemolyticus.* The figure was generated with ESPript. *F*, the fluctuation of RMSD values of VP14460 and VP14460-VP14465pep complex.*G*, the structural sizes using the gyration radius (Rg) in VP14460 and VP14460-VP14465pep complex. *H*, R.m.s.f. of each amino acid in VP14460 and VP14460-VP14465pep complex. VP14460 (*blue*) and VP14460-VP14465pep complex (*red*) are shown.

Since the α_2_/α_3_ region of VP14465 is the primary binding site for VP14460, to explore the molecular mechanism underlying the neutralization of toxin VP14465 by VP14460, we determined the crystal structure of the VP14460-VP14465pep (residues 394–416) complex at a resolution of 2.26 Å. The electron density map for the bound peptide was well resolved, allowing unambiguous placement of the peptide (Fig. 5*A*). The well-resolved and distinguishable substrate-binding pocket (Fig. 5*B*), predominantly composed of hydrophobic residues, primarily including Asp, Arg and Lys, are formed by α2, α3, α5, and the loop connecting α9 and α10 of VP14460 (Fig. 5*C*). Specifically, the N-terminal and C-terminal ends of the peptide are anchored by three pairs of hydrogen bonds with R52, W55, and S108 of VP14460, respectively. The intermediate interaction network involves three consecutive amino acids from the peptide engaging with D107 in α5 of VP14460, as well as D209, R215, and T222 in the loop between α9 and α10. The final set of interactions comprises two hydrogen bonding forces formed between the side chain hydroxyl group of Y28 from VP14460 and L407- E411 of VP14465, respectively (Fig. 5*D*). The amino acids responsible for binding and neutralizing toxin VP14465 exhibit substantial conservation across different genera of the *Vibrio* genus (Fig. 5*E*, with asterisks). This conservation pattern strongly suggests that the neutralization mechanism of this E-I module is evolutionarily conserved and likely operates through similar molecular pathways across diverse *Vibrio* species.

Although no significant difference was observed in the crystal structure of VP14460 upon peptide binding (RSMD = 0.246 Å) (Fig. 4*C*), molecular dynamics (MD) simulations revealed distinct dynamic behaviors between the two structures. Throughout the 200 ns simulation, the complex (final RMSD = 0.223 nm) was more stable than the protein alone (final RMSD = 0.274 nm), exhibiting only minor fluctuations (Fig. 5*F*). The radius of gyration (Rg) indicated that the complex was slightly less compact than the protein alone, with Rg values ranging from 2.0 to 2.1 nm (final 2.032 nm) and 1.9 to 2.1 nm (final 2.007 nm), respectively, over the 200 ns simulation (Fig. 5*G*). Root mean square fluctuation analysis of individual amino acid positions demonstrated globally reduced mobility in the bound complex (Fig. 5*H*). Specifically, the fluctuations of amino acid residues 20 to 60, critical for peptide binding, were markedly attenuated, with most residues in this range showing decreased mobility, this is consistent with the observed interactions between Y28, K45, R52, W55 of VP14460 and peptides from a structural perspective. Overall, the complex exhibited lower RMSD and root mean square fluctuation values than the free protein, indicating reduced conformational fluctuations and enhanced stability upon ligand binding. Despite a slightly higher Rg value, the complex remained stable throughout the simulation, suggesting a more expanded yet stable conformation.

### VP14465 contains a DNA-binding pocket

**Figure 6:**
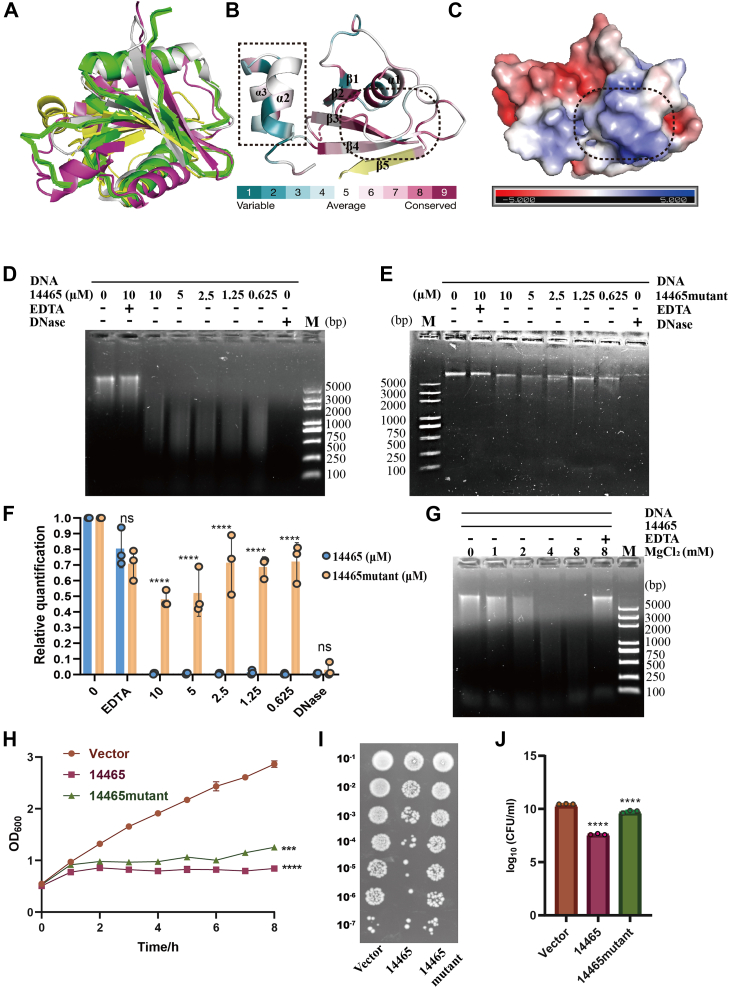
**A potential DNA-binding Pocket in VP14465.***A*, superposition of VP14465C (*yellow*) with the highest-scoring Dali search structures, Protein Data Bank codes 8HMY, 1A79, 3AJV, and 6Z9U, depicted in magenta, *green*, *gray*, and *orange*, respectively. *B*, cartoon representation and ConSurf analysis of the VP14465C structure using nine-color conservation scores. *C*, the electrostatic surface potential of VP14465C structure (*red, blue* and *white* indicate negative, positive and neutral potentials, respectively). The two modules are marked with a rectangular dashed box and a circular dashed box respectively. *D* and *E*, nuclease activity of VP14465 and its mutant. VP14465 mutant includes residues involved in DNA interactions, including W396, R400, Y354, R355 and T356. Plasmid DNA (pET28a) was used as the substrate. All reactions were conducted at 37 °C for 30 min. Experiments were performed in triplicate, yielding consistent results, with representative data shown. *F*, relative quantitation of nuclease activity of VP14465 and its mutant. *G*, nuclease activity of VP14465 at different MgCl_2_ concentrations (0, 1, 2, 4, 8 mM). *H*, the effects of VP14465 and its mutant production on the culture density of *E*. *coli* strains. Culture densities were enumerated by OD_600_ at subsequent time points. *I* and *J*, the survival of *E. coli* cells was examined by serial dilution by dilution spot tests. All cultures were grown in LB medium and induced with IPTG at time zero with an OD_600_ value of 0.5. Record the OD_600_ value of the bacterial suspension once per hour. After further incubation, cells were plated to enumerate CFU. Each point represents the average of triplicate values, *error bars* represent standard deviation. The growth curves and CFU assay results of VP14465 and its mutants were compared with those of the vector control group using one-way ANOVA. Statistical significance is indicated as follows: ns, not significant, ∗*p* < 0.05, ∗∗*p* < 0.01, ∗∗∗*p* < 0.001, ∗∗∗∗*p* < 0.0001. CFU, colony-forming unit.

In the electron density map of the C-terminal domain of VP14465 (comprising 146 amino acids), only residues 301 to 353 and 392 to 437 exhibited clear visibility. The topological architecture of VP14465C displays a circularly permuted fold with the secondary structure arrangement: NH_2_-α_1_β_1_β_2_β_3_α_2_α_3_β_4_β_5_-COOH. Based on the structure-based multiple sequence alignment of the top 100 homologs in a Dali search, the comparison with PDB showed that the Z-score did not exceed 7.1, the results indicate that VP14465 may have similar functions to tran-splicing endonuclease (Fig. 6*A*). Structurally, VP14465C can be divided into two functional modules, the α_2_/α_3_ module (marked with a rectangular dashed box), which harbors a negatively charged surface, is presumably responsible for binding to VP14460 (Fig. 6, *B* and *C*). The spherical α_1_-β_1_-β_2_-β_3_-β_4_-β_5_ module (marked with a dashed circular box) contains highly conserved amino acids, including the previously identified Kx_3_GEx_n_GIDx_2_Yx_n_Yx_3_ExK motif (Figs. 6*B* and S4*A*). This conserved motif resides at the core of the three β-sheets, forming a positively charged pocket in the central region (Fig. S4*B*), which may mediate DNA binding interactions. Finally, we docked the DNA molecule to the putative binding pocket in VP14465 using the C-terminal domain structure by AlphaFold3 (with ipTM = 0.63, pTM = 0.77). This complex reveals that DNA is anchored at both ends. One end is secured through hydrogen bonds formed by the side chains of W396 and R400 at the 14465C end (Fig. S4*C*), which has been identified as the primary binding site for VP14460. The opposite end is stabilized by three amino acids: Y354, R355, and T356 (Fig. S4*D*), a region where electron density is absent in the VP14465C structure in this study.

Next, the potential nuclease activity of VP14465 was assessed by monitoring its ability to cleave plasmid DNA (pET28a). Purified VP14465C exhibited bivalent metal ion-dependent DNase activity *in vitro* in a concentration-dependent manner (Fig. 6*D*). Mutation of all aforementioned 5 residues to alanine (14465 mutants) resulted in a marked reduction in DNA cleavage activity (Fig. 6*E*). Relative quantitative analysis further revealed that the WT VP14465C was able to completely degrade DNA at a concentration as low as 0.625 μM. In contrast, the mutation exhibited severely impaired DNase activity, achieving only 34.67% DNA degradation even at the highest concentration tested (10 μM), although a concentration-dependent trend was still observable (Fig. 6*F*). We next investigated the metal ion dependency of the DNase activity. The activity of VP14465C increased progressively with rising Mg^2+^ concentrations, achieving complete DNA degradation at 4 mM Mg^2+^ (Figs. 6*G* and S4*E*). Furthermore, the DNase activity was specifically dependent on Mg^2+^ and Mn^2+^, but not on Ca^2+^, Cu^2+^, Zn^2+^, or Ni^2+^ (Fig. S4, *F* and *G*).

Consistently, *in vivo* assays revealed impaired growth of *E. coli* expressing the VP14465 mutant strain (Fig. 6*H*), a finding further corroborated by spot dilution assays evaluating bacterial survival rates through serial dilution (Fig. 6, *I* and *J*). The discrepancy between OD_600_ and CFU assays (Fig. 6, *H*–*J*) reflects two factors. First, VP14465 is a DNA-damaging toxin that kills bacteria without causing lysis, so dead cells remain intact and maintain OD_600_. Second, the growth curve was measured with continuous inducer, whereas CFU assay was performed on inducer-free plates, where toxin expression ceased and surviving bacteria resumed growth. To further validate this mechanism, we extracted genomic DNA from control and experimental groups. Compared with the control and the mutant strain, genomic DNA from the VP14465-expressing strain appeared dispersed and degraded, confirming that the toxin exerts its bactericidal effect through genomic DNA cleavage (Fig. S4*H*).

Actually, the α_2_/α_3_ module of VP14465 constitutes the overlapping region responsible for both toxin neutralization and DNA binding (Fig. S4*E*, marked with a rectangular dashed box). Functioning as an E-I pair, it acts as the core binding center for the neutralization of toxins by VP14460. Moreover, as a toxin secreted *via* T6SS, this module plays a critical role in binding to and subsequent degradation of DNA, a process that directly enables the killing of competing bacterial strains.

### Essential residues for neutralizing VP14465 toxicity

**Figure 7:**
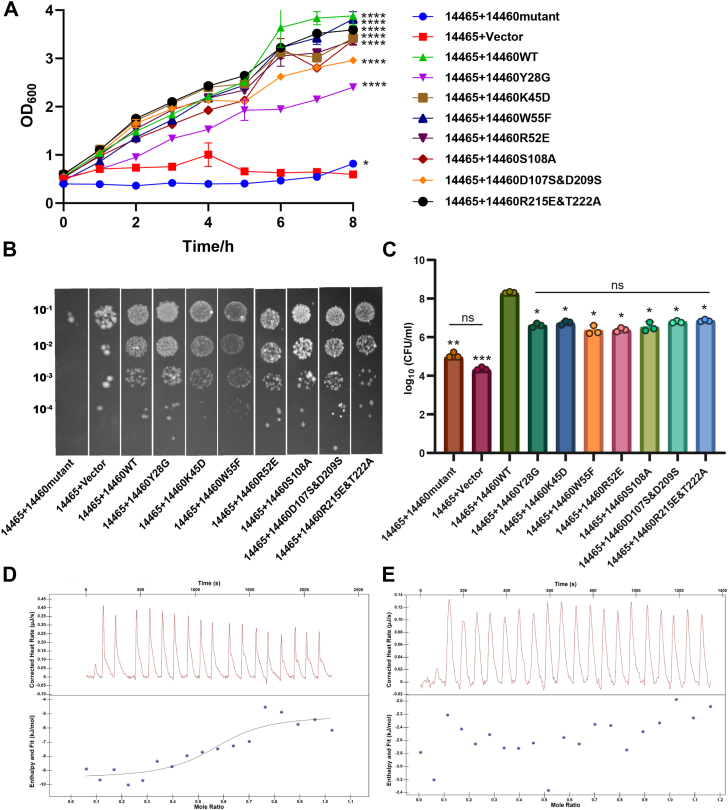
**The essential amino acid residues in VP14460 that neutralize VP14465 toxicity.** Site-directed mutagenesis of VP14460 was done, as described in Materials and methods, and mutations were identified by DNA sequence determination. *A*, effects of the co-expression of VP14465 and VP14460 variants on the cell growth of *E. coli*. Culture densities were enumerated by OD_600_ at subsequent time points. All cultures were grown in LB medium and induced with IPTG at time zero with an OD_600_ value of 0.5. Record the OD_600_ value of the bacterial suspension once per hour. *B*, growth of *E. coli* in agar plates harboring a vector co-expressing VP14465 and VP14460 variants. The cells were prepared with serial 10-fold dilutions from top to *bottom*. *C*, cell viability assay. Coexpression of VP14465 and VP14460 variants abolishes inhibition. Each point represents the average of triplicate values, error bars represent standard deviation. Growth curve and CFU assays between VP14460 and its variants were compared and analyzed using one-way ANOVA. Statistical significance is indicated as follows: ns, not significant, ∗*p* < 0.05, ∗∗*p* < 0.01, ∗∗∗*p* < 0.001, ∗∗∗∗*p* < 0.0001. *D*, ITC of VP14465 peptide and VP14460 interaction at 25 °C. *E*, ITC of VP14465 peptide and VP14460 binding-site mutant interaction at 25 °C. The bottom graph illustrates the integrated heat for each injection of VP14465 peptide together with a fit, whereas the y axis represents the heat released per mole for each injection. CFU, colony-forming unit.

To validate the functional relevance of the binding sites identified in our crystal structure, we employed isothermal titration calorimetry (ITC) and *in vivo* bacterial viability assays. Prokaryotic expression plasmids harboring VP14460 (or its variants) and VP14465 with distinct resistance markers were co-transformed into *E. coli*, and their effects on bacterial growth kinetics were monitored. To provide a clear spatial context of the interaction interface, the residues mutated on VP14460 have been mapped onto the structural model (Fig. S5). As shown in Figure 7*A*, bacterial growth inhibition was comparable to that in the control group only when all interacting amino acids in VP14460 were simultaneously mutated (14460mutant), with merely a ∼0.22 log reduction in optical density at 8 h post-induction, indicating full neutralization of VP14465-mediated toxicity. In contrast, single-point mutations in VP14460 failed to eliminate VP14465 toxicity, as their growth curves closely resembled that of the strain cotransformed with WT VP14460. Notably, the WT VP14460 cotransformed strain achieved the highest OD_600_ due to full neutralization of VP14465 toxicity. Among all single mutants, the Y28G substitution in VP14460 led to slightly impaired toxicity-neutralizing capacity, causing a log reduction of ∼1.48 relative to the group co-expressing VP14465 and 14460mutant. We further validated the bactericidal activity of the toxin and the neutralizing capacity of the immunity *via* gradient dilution spot plating assay, and the results were consistent with those of bacterial growth kinetics analysis (Fig. 7*B*). Quantitative analysis of viable bacteria revealed that the fully mutated VP14460 with all binding sites were disrupted markedly elevated cytotoxicity which was comparable to that of the toxin-only control group. This effect is attributable to the loss of its capacity to bind VP14465, ultimately resulting in a 3.31-log reduction in bacterial viability (no significant difference between the two groups). In contrast, single-site mutations in VP14460 only partially attenuated VP14465-mediated toxicity, yielding a 1.42 to 1.93 log reduction in viable counts (Fig. 7*C*). ITC revealed that the dissociation constant (K_d_) between WT VP14460 and VP14465 peptide was 4.76 nM (Fig. 7*D*). In contrast, no detectable binding affinity was observed for the VP14460 binding-site mutant (Fig. 7*E*). Although single-point mutants of VP14460 still exhibited measurable binding affinity to the peptide, their binding capacity was significantly weaker than that of the WT (Table 1, Fig. S6). Collectively, these results demonstrate that individual binding interactions contributing to the association between VP14460 and VP14465, and thus the neutralization of VP14465 toxicity, exhibit distinct contributions to the overall binding affinity. Complete abrogation of their interaction requires simultaneous mutation of all critical amino acid residues involved in the binding interface.

**Table 1:** Kinetics and affinity constants for WT and VP14460 mutants binding to VP14465 peptide

VP14460	Kd(M)	Enthalpy changeΔH (kJ/mol)	Entropy changeΔS (J/mol·K)
WT	(4.76 ± 0.27) × 10^−9^	−28.99	6.21 × 10^1^
Y28G	(2.80 ± 0.91) × 10^−9^	−5.23	1.46 × 10^2^
K45D	(6.37 ± 2.33) × 10^−8^	−0.48	1.36 × 10^2^
R52E	(1.19 ± 0.58) × 10^−6^	−2.33	1.06 × 10^2^
W55F	(3.39 ± 2.00) × 10^−7^	−21.78	5.08 × 10^1^
D107S-D209S	(1.31 ± 2.24) × 10^−7^	−11.86	2.22 × 10^2^
S108A	(1.08 ± 0.43) × 10^−8^	−0.34	1.51 × 10^2^
R215E-T222A	—	—	—
Mutants	—	—	—

## Discussion

Our findings demonstrate that the endonuclease effector VP14465 and its cognate immunity VP14460 constitute a functional E-I module in *V. parahaemolyticus*. Consistent with E-I module behavior, heterologous expression of the *VP14465* toxin gene in *E*. *coli* resulted in significant growth inhibition, which was effectively neutralized by co-expression of the *VP14460* immunity. To elucidate the molecular basis of toxin neutralization, we determined the first high-resolution crystal structures of both the standalone VP14460 immunity and the VP14460-VP14465 E-I complex. These structural insights provide a foundation for understanding the mechanistic details of toxin inhibition and regulatory interactions within this E-I module.

VP14460 exists as a dimer in its free state but forms a monomeric complex when bound to the toxin VP14465. In contrast to the majority of E-I modules, only a subset employs conformational remodeling upon complex formation as their molecular strategy. Examples include the Tae4-Tai4 complex (a compact heterotetramer, whereas Tai4 exists as an all-helical protein that forms a dimer in solution (40)) and the *Staphylococcus aureus* PemI antitoxin, which neutralizes the PemK toxin dimer by inducing structural rearrangements (41, 42). Our structural analysis reveals that VP14460 maintains a relatively stable conformation upon binding VP14465. This difference may be attributed to VP14460’s dimeric state and its distinct interaction interface with VP14465. Unlike the *Legionella* effector SdeA, a typical ADP-ribosyltransferase toxin that undergoes significant conformational changes upon ubiquitin binding (43), or the PemI-PemK system, where the antitoxin (PemI) spatially occludes the active site and RNA-binding residues of the toxin (PemK) (also leading to substantial structural changes) (41), the VP14460-VP14465 complex exhibits minimal conformational alteration. This suggests an alternative neutralization mechanism, possibly relying on interface-specific inhibition rather than large-scale structural remodeling.

Notably, the R52E mutant exhibited a significantly reduced Kd value compared with other mutants in the ITC assay (Table 1), yet this difference was not visually apparent in the OD_600_ growth curves (Fig. 7*A*). This discrepancy likely reflects the different sensitivities of the two assays. ITC directly quantifies binding affinity in a purified *in vitro* system, whereas OD_600_ growth curves integrate multiple *in vivo* processes (*e.g.*, protein expression, folding, stability, and complex formation) under continuous induction. Consequently, moderate differences in binding affinity may not translate into discernible differences in bacterial growth. Nevertheless, all tested mutants showed significantly impaired function compared with WT VP14460 in both assays, confirming the importance of the identified interfacial residues.

VP14465 consists of two distinct domains, with bioinformatics analysis using the XANNpred Server (44) predicting substantial disordered regions between them. Initially, we pursued crystallographic determination of the full-length VP14465 structure. Despite multiple crystallization attempts, we were only able to obtain crystals of the C-terminal domain, which was isolated through pull-down assays *via* its interaction with VP14460. Interestingly, the prokaryotic expression protein of VP14465 was further purified *via* size exclusion chromatography, yielding three concurrent elution peaks (Fig. S7*A*). Their molecular weights corresponded to the oligomeric (potentially octameric), dimeric, and monomeric forms of VP14465, respectively. Western blot analysis using His-tag-specific antibodies demonstrated consistent reactivity across all three samples (Fig. S7*B*). These findings suggest that VP14465 likely functions in an oligomeric state, implying that cryo-electron microscopy (cryo-EM) would be particularly suitable for determining the three-dimensional structure of its full-length form or its C-terminal domain (VP14465C). Based on the Superdex 75 elution profile (Fig. S7*A*), which suggested an oligomeric state between octamer and hexadecamer (158–440 kDa), AlphaFold3 was used to predict multimeric assemblies of VP14465. Among the tested models (octamer to hexadecamer), only the octamer showed high confidence (ipTM = 0.63, pTM = 0.77) and was selected for further analysis. Structural analysis reveals that the C-terminal domain is organized into a central ring, a architecture that may function as the substrate-binding site. (Fig. S7*C*).

Collectively, we propose a model for the action mechanism of the VP14460-VP14465 E-I module delivered *via* the T6SS during bacterial competition (Fig. S7*D*). Under non-stress conditions, the toxin VP14465 interacts with the immunity VP14460 in a monomeric state. However, upon exposure to competitive threats, VP14465 is released and oligomerizes, while VP14460 forms dimers. Concurrently, bacteria deploy toxic effectors through T6SSs to outcompete rival cells. Specifically, VP14465 acts as a genome-binding toxin that degrades nucleotides, ultimately inducing prey cell death. The monomer-dimer transition of VP14460 is critical for releasing active VP14465, which subsequently oligomerizes to exert nuclease activity. While our biochemical and structural data support a model in which VP14460 sequesters VP14465 as a monomer and release allows VP14465 to oligomerize, direct evidence for a toxin-induced conformational transition (*e.g.*, by hydrogen-deuterium exchange mass spectrometry or single-molecule FRET) is currently lacking. Addressing this question will be an important focus of our future research.

## Experimental procedures

### Protein expression and purification

A codon-optimized gene of *vp14460* was synthesized (Tsingke Biotech) and inserted into a modified pRSFDuet-1 expression vector (Novagen) with 6 × His tag at N-terminus (marked as RSF-14460 in text). VP14465 C-terminal peptide (residues 394–416, VP14465pep) and residues 292 to 499 (VP14465C) was amplified and cloned into the pGEX-6p-1 (Novagen) vector with a GST tag and a PreScission protease cleavage site. The VP14460-VP14465C and VP14460-VP14465pep protein complexes were produced through co-expression in *E. coli* BL21 (DE3) competent cells (Novagen) *via* co-transformation of dual plasmids carrying ampicillin and kanamycin resistance markers. Following transformation, bacterial cultures were grown in LB broth supplemented with both antibiotics at 37 °C with shaking (220 rpm) until mid-log phase (OD_600_ = 0.6), at which point protein expression was induced by 0.3 mM IPTG with subsequent incubation at 16 °C for 20 h. After lysis, sonication and centrifugation, the clarified cell lysate of VP14460 and complex proteins were incubated with nickel Sepharose affinity resin (GE Healthcare) and glutathione-Sepharose beads (GE Healthcare), respectively. VP14460 protein was eluted with elution buffer (25 mM Tris-HCl pH 8.0, 150 mM NaCl, 300 mM imidazole, 1 mM PMSF), while the untagged complex proteins were obtained after the beads were incubated with PreScission protease at 4 °C. Proteins were further purified by size exclusion chromatography on a Superdex 75 Increase 10/300 Gl column (GE Healthcare) that had been pre-equilibrated in a buffer of 25 mM Tris·Cl pH 8.0, 150 mM NaCl, 1 mM DTT.

### Crystallization and structure determination

All the purified proteins were crystallized by vapor diffusion in sitting-drops with a 1:1 mixture of sample and reservoir solution. Crystallization conditions were screened using commercial kits from Hampton Research and Qiagen (JCSG). The VP14460 crystals used for data collection were appeared using sitting drop vaporization method in a reservoir solution containing (0.2 M sodium tartrate dibasic dihydrate, 20% w/v polyethylene glycol 3350), while the complex VP14460-VP14465C and VP14460-VP14465pep crystals appeared in solution (0.2 M potassium chloride, 20% w/v polyethylene glycol 3350) and (1.0 M sodium citrate, 0.1 M CHES pH 9.5), respectively. Diffraction data were collected on the BL19U1 station at the Shanghai Synchrotron Radiation Facility (SSRF). Images were recorded at 0.987 Å wavelength with 1^◦^ oscillation step and 0.5 s exposure time per frame, and then were processed using XDS software program (https://xds.mr.mpg.de). The initial model of VP14460 was solved by molecular replacement using the structure predict by Alphafold3 as the searching model, while the complex proteins was solved by molecular replacement by the solved VP14460 structure. The structures were rebuilt with Phenix and Coot, and refined using Phenix and REFMAC. The orientations of the amino acid side chains and bound water molecules were modeled on the basis of 2Fobs_Fcalc and Fobs_Fcalc difference Fourier maps. Electron density maps (2Fo-Fc) were calculated using PHENIX and visualized in PyMOL. Maps were contoured at 1.0 σ for figure preparation. All structural molecular graphics were generated with UCSF Chimera and PyMOL. The Table S1 shows detailed data collection and refinement statistics. The structures presented in this paper have been deposited in PDB with the code: 9VY2, 9WAQ and 9VYD.

### Pull down

For the GST pull-down assay, GST-tagged proteins (VP14465pep or VP14465C) were immobilized on glutathione-Sepharose 4B beads (Cytiva) and incubated with 6 × His-tagged VP14460 in binding buffer (20 mM Tris-HCl, pH 8.0, 150 mM NaCl, 1 mM DTT) at 4 °C for 2 h. After extensive washing, bound proteins were eluted with 10 mM reduced glutathione in elution buffer (50 mM Tris-HCl, pH 8.0). The eluates were analyzed by SDS-PAGE followed by Coomassie Blue staining.

### Isothermal titration calorimetry

ITC experiments were conducted on an Affinity ITC system (TA Instruments) maintained at 25.0 ± 0.1 °C. The sample chamber (cell volume = 190 μl) contained 400 μM VP14465pep dissolved in phosphate-buffered saline (pH 7.4). Titrant solutions (syringe volume = 40 μl) consisted of 100 μM WT VP14460 or its variants. The injection protocol comprised 20 successive aliquots (2.0 μl per injection) with 1 s injection duration, 150 s equilibration intervals, and constant stirring at 200 rpm. Thermodynamic parameters were derived through nonlinear regression analysis using Origin software (OriginLab Corporation, https://www.originlab.com/) with the single-site binding model. All ITC measurements were performed in triplicate (three independent experiments). The Kd values are reported as mean ± SD calculated from the three replicates.

### Bacterial growth inhibition and bactericidal activity assays

To analyze the growth inhibition rates, the plasmids pGEX-VP14465C and RSF-VP14460 or its variants were co-transformation into *E. coli* BL21 (DE3) competent cells and then co-expression at an OD_600_ value of 0.5, then the OD_600_ values of the bacterial suspension were recorded every hour. For plaque assay, after expression for 20 h at 16 °C, tenfold serial dilutions of the bacteria suspensions were prepared in phosphate-buffered saline (10 mM, pH 7.4), and 1 μl aliquots were spotted onto LB solid culture medium. For bactericidal assay, the residual CFU of bacterial suspensions was calculated by plating serial dilutions on agar. Finally, 100 μl of the diluted sub-cultured suspensions were spread on the solid LB agar plate, and colonies formed after 20 h incubation at 37 °C were counted. All experiments were conducted in biological triplicate.

### Gel-based DNase activity assay

DNase activity was assessed by monitoring the cleavage of plasmid DNA. Each reaction mixture (20 μl) contained 300 ng of pET28a plasmid DNA (Novagen), 4 mM MgCl_2_, 5 mM EDTA, and reaction buffer (10 mM Tris-HCl, 50 mM NaCl, pH 8.0), supplemented with purified VP14465 protein at final concentrations of 0, 0.625, 1.25, 2.5, 5, or 10 μM. Reactions were incubated at 37 °C for 30 min, then mixed with 10 × loading buffer to a final concentration of 1 × . Samples were electrophoresed on a 0.6% agarose gel prepared in TBE buffer containing Gel Red, at 140 V for 30 min.

### Statistical analysis

The bacterial growth rates and bactericidal activity data were evaluated by one-way ANOVA, followed by appropriate *post hoc* tests where applicable. Results are presented as mean ± SD. Statistical significance was defined as follows: *p* < 0.05 (∗), *p* < 0.01 (∗∗), *p* < 0.001 (∗∗∗), and *p* < 0.0001 (∗∗∗∗), while non-significant (ns) differences were indicated for *p* ≥ 0.05.

### APBS-based electrostatic potential calculation

Electrostatic surface potentials were calculated using the APBS (Adaptive Poisson-Boltzmann Solver, Version 3.0.0) plugin within PyMOL. The protein structures were prepared using PDB2PQR to assign partial charges and add missing hydrogen atoms. APBS calculations were performed with the following parameters: dielectric constant of solute = 2.0, dielectric constant of solvent = 78.0, temperature = 25 °C, and ion concentration = 0.15 M NaCl. The electrostatic potential was mapped onto the solvent-accessible surface, and the color scale was set uniformly to −5 to +5 kT/e for all figures.

### Molecular-dynamics simulations

The GROMACS2021.5 software package (https://www.gromacs.org/) is utilized for conducting MD simulations of protein-ligand interactions, employing the Amber14sb force field. The simulations incorporate the TIP3P water model as the primary solvent model, with periodic boundary conditions applied. The simulation protocol comprises four main stages: energy minimization, NVT ensemble equilibration, NPT ensemble equilibration, and production dynamics simulation. Initially, heavy atoms of the VP14460-VP14465pep complex are restrained to optimize the water molecule energies through 500 steps of steepest descent minimization. Subsequently, a 50,000-step NVT ensemble simulation is conducted while maintaining the constraints, with a temperature of 25 °C and a time step of 2 fs. Following this, a 50,000-step NPT ensemble simulation is carried out for the entire system at 25 °C with a time step of 2 fs. Finally, a 200 ns MD simulation in the NPT ensemble with a time step of 2 fs is performed. The analysis of the simulation parameters is conducted using the GROMACS software package.

## Data availability

The structure presented in this paper has been deposited in PDB with the codes: 9VY2, 9WAQ and 9VYD.

## Supporting information

This article contains supporting information (34, 37).

## Conflict of interest

The authors declare that they have no conflicts of interest with the contents of this article.
