# Supplementary material for: Structural and mechanistic insights into the VP14460-VP14465 effector-immunity module of the Vibrio parahaemolyticus type VI secretion system
Source: J Biol Chem. 2026 Jun 15;302(8):113257. doi: 10.1016/j.jbc.2026.113257 (PMC13377157; doi:10.1016/j.jbc.2026.113257)
Supplement: Supporting Figures and Table [file mmc1.doc]

*Supporting information*

**Structural and mechanistic insights into the VP14460-VP14465 effector-immunity module of the***Vibrio parahaemolyticus***type VI secretion system**

Yuyan Zheng^1#^, Chenhua Zheng^1#^, Zhouyang Ye^1#^, Lirui Huang^1^, Xuezhongqing Lin^1^, Binjie Wu^1^, Zihang Pan^1^, Rui Qiu^1^, Jinyuan Cai^1^, Linchen Xu^1^, Zilin Deng^1^, Ruxin Xu^1^, Xi Xie^2,3*^, Lihua Xie^1*^ and Fen Hu^1*^

^1^Key Laboratory of Ministry of Education for Gastrointestinal Cancer, School of Basic Medical Sciences, Fujian Medical University, Fuzhou 350122, China

^2^State Key Laboratory of Membrane Biology, MOE Key Laboratory of Bioinformatics, Tsinghua-Peking Center for Life Sciences, School of Life Sciences, Tsinghua University, Beijing, China

^3^State Key Laboratory of Drug Research, Shanghai Institute of Materia Medica, Chinese Academy of Sciences, Shanghai, 201203, China

# Yuyan Zheng, Chenhua Zheng and Zhouyang Ye contributed equally to this work.

^*^To whom correspondence should be addressed. Email: [fenhu@fjmu.edu.cn](mailto:fenhu@fjmu.edu.cn); [lhxie@fjmu.edu.cn](mailto:lhxie@fjmu.edu.cn); 513367167@qq.com

**Table S1. Data collection and refinement statistics of proteins**.

| **Parameters** | **VP14460** | **VP14460-VP14465C** | **VP14460-VP14465pep** |
| --- | --- | --- | --- |
| **Data collection** |  |  |  |
| Wavelength (Å) | 0.9793 | 0.9793 | 0.9793 |
| Space group | *C* 1 2 1 | *P* 1 | *P* 4_3_ 2_1_ 2 |
| Cell dimensions |  |  |  |
| *a, b, c* (Å) | 117.83, 89.08, 82.81 | 53.84, 58.48, 80.13 | 97.08, 97.08, 107.32 |
| α, β, γ (°) | 90, 118.26, 90 | 89.90, 89.84, 62.92 | 90, 90, 90 |
| Resolution range (Å) | 28.55-2.32 (2.40-2.32) | 30.72-1.97  (2.04- 1.97) | 57.83 - 2.5  (2.59 - 2.5) |
| Unique reflections | 29639 (2617) | 57967 (5555) | 16207 (1339) |
| Multiplicity | 3.2 (3.3) | 5.9 (6.0) | 24.8 (25.3) |
| Completeness (%) | 99.8 (99.9) | 99.99 (100) | 99.7 (100) |
| Mean I/sigma I | 6.2 (1.7) | 3.5 (1.7) | 10.4 (1.4) |
| Wilson B-factor (Å^2^) | 27.12 | 23.61 | 30.62 |
| Rmerge | 0.087 (0.590) | 0.377 (1.211) | 0.398 (3.046) |
| **Refinement** |  |  |  |
| Reflections used in refinement | 29630 (2616) | 57960 (5555) | 16196 (1339) |
| Reflections used for R_free_ | 1804 (152) | 2914 (256) | 1618 (134) |
| Rwork | 0.1983 (0.2873) | 0.2120 (0.2660) | 0.2243 (0.3152) |
| Rfree | 0.2504 (0.3602) | 0.2521 (0.3024) | 0.2685 (0.3804) |
| Number of atoms |  |  |  |
| Macromolecules | 5241 | 6881 | 2858 |
| Ligands | 0 | 62 | 0 |
| Solvent | 233 | 554 | 61 |
| Rmsd from ideal |  |  |  |
| Bond lengths (Å) | 0.003 | 0.011 | 0.003 |
| Bond angles (°) | 0.65 | 1.28 | 0.63 |
| Ramachandran plot |  |  |  |
| Favored (%) | 97.47 | 98.19 | 98.27 |
| Allowed (%) | 2.37 | 1.45 | 1.45 |
| Outliers (%) | 0.16 | 0.36 | 0.29 |
| Average B-factor | 36.65 | 28.45 | 37.23 |
| Clashscore | 5.04 | 5.06 | 5.70 |
| PDB code | 9VY2 | 9WAQ | 9VYD |

*^a^* Statistics for the highest-resolution shell are shown in parentheses.


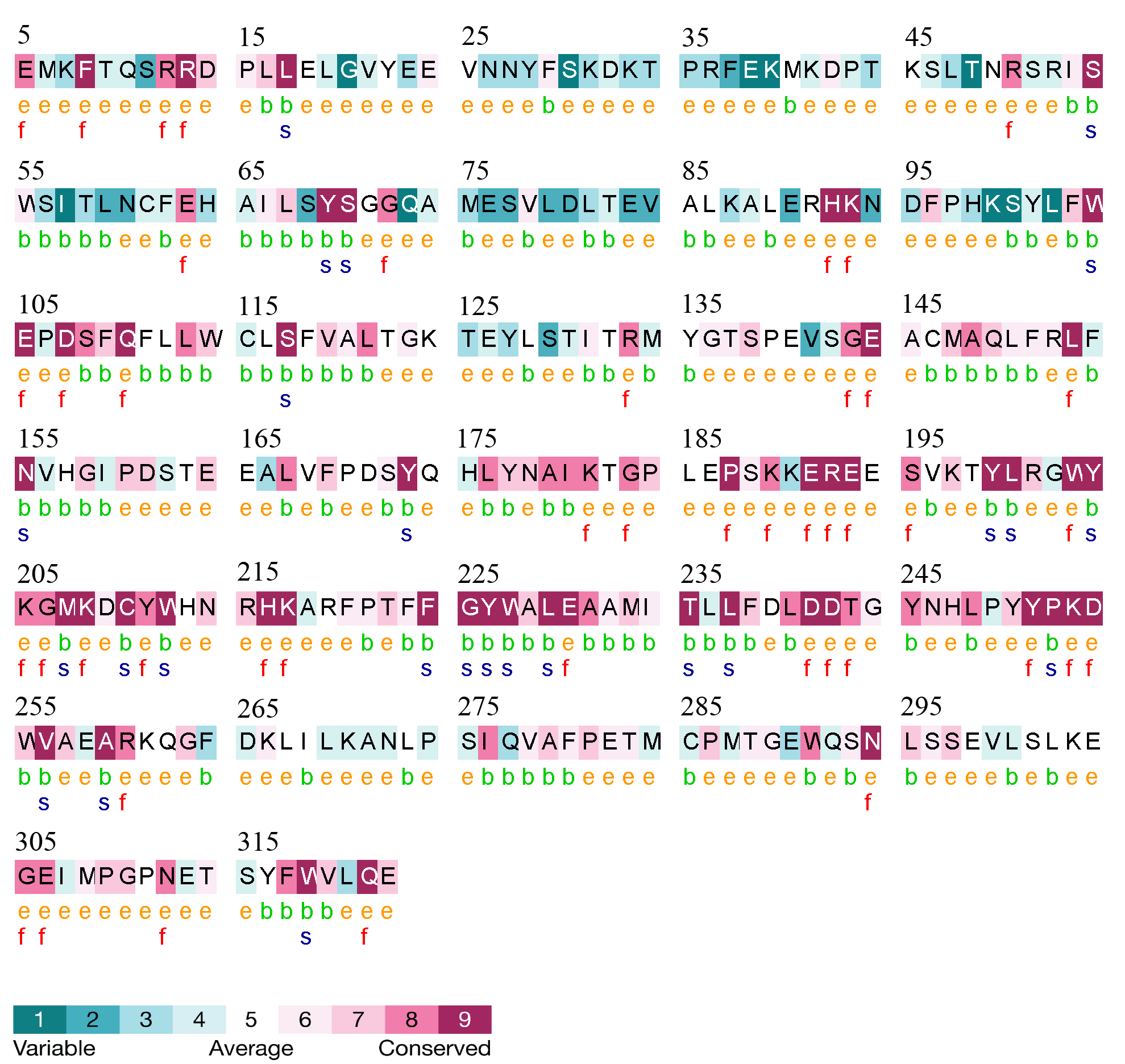


**Figure S1. ConSurf analysis of the VP14460 sequence using nine-color conservation scores.** e - An exposed residue according to the neural network algorithm. b - A buried residue according to the neural network algorithm. f - A predicted functional residue (highly conserved and exposed). s - A predicted structural residue (highly conserved and buried).


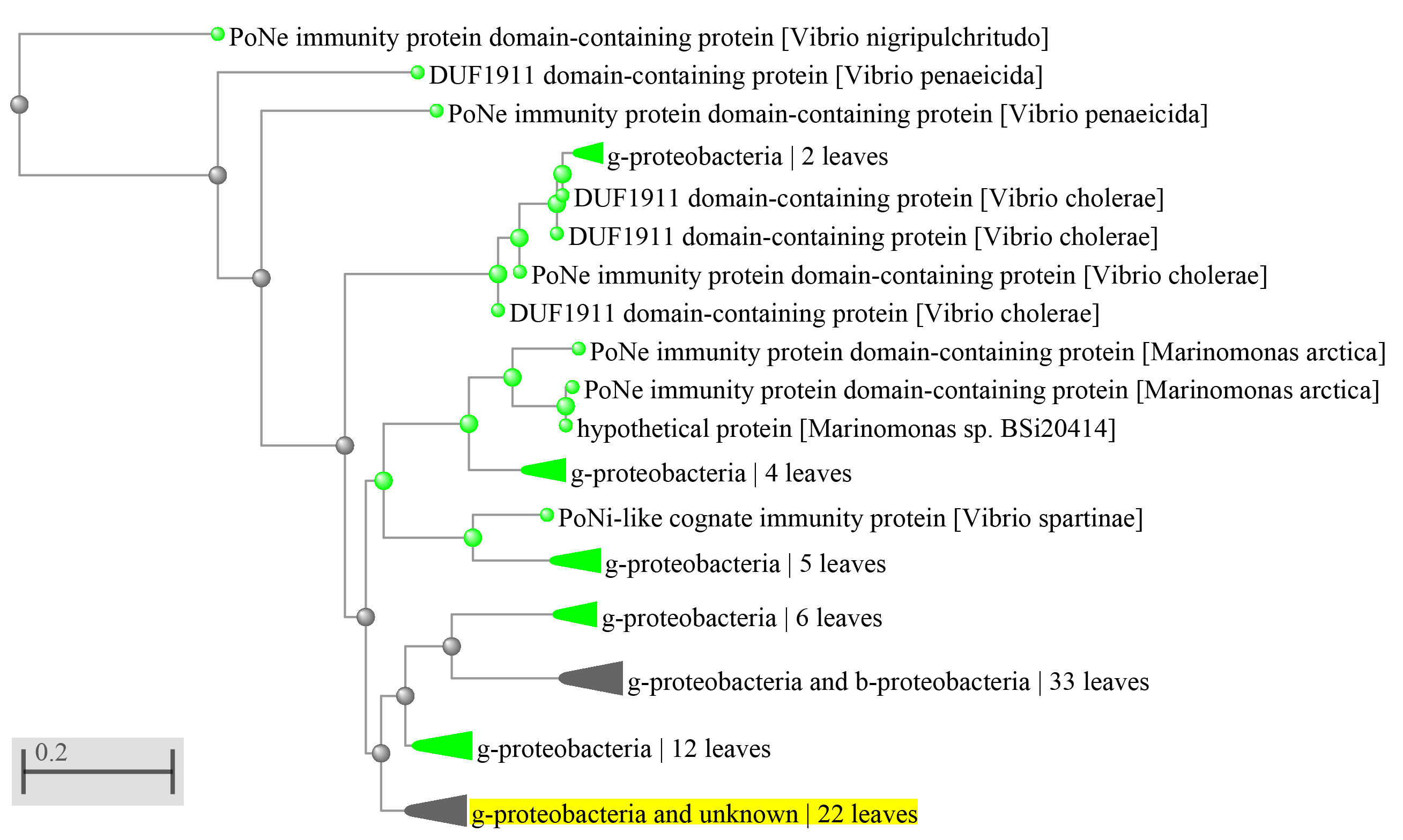


**Figure S2. Phylogenetic distribution of the DUF1911 domain across species as identified by Dali server analysis.**

**
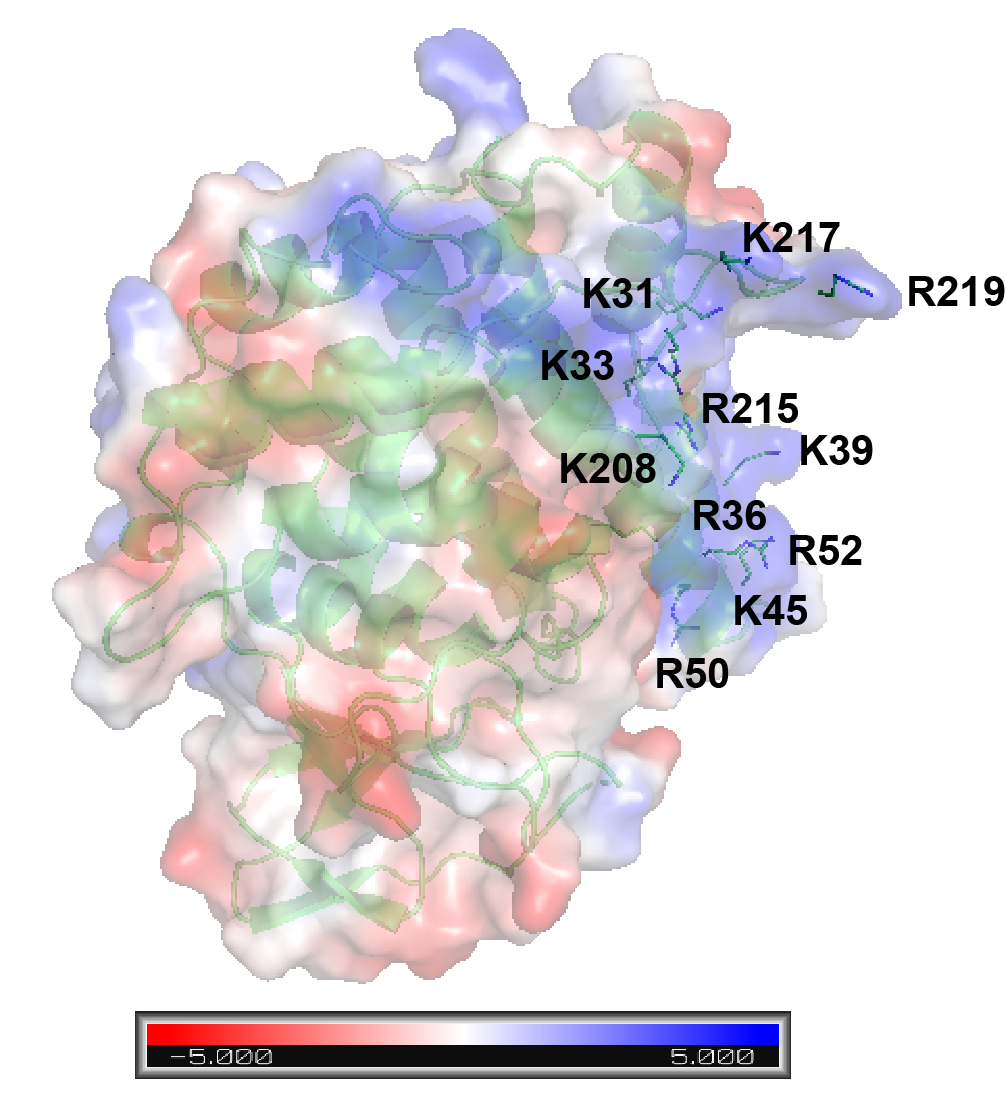
**

**Figure S3. Electrostatic surface potential of VP14460.** The molecular surface is colored according to electrostatic potential (red, blue and white indicate negative, positive and neutral potentials, respectively). Positively charged residues surrounding the binding pocket are highlighted as sticks.

**
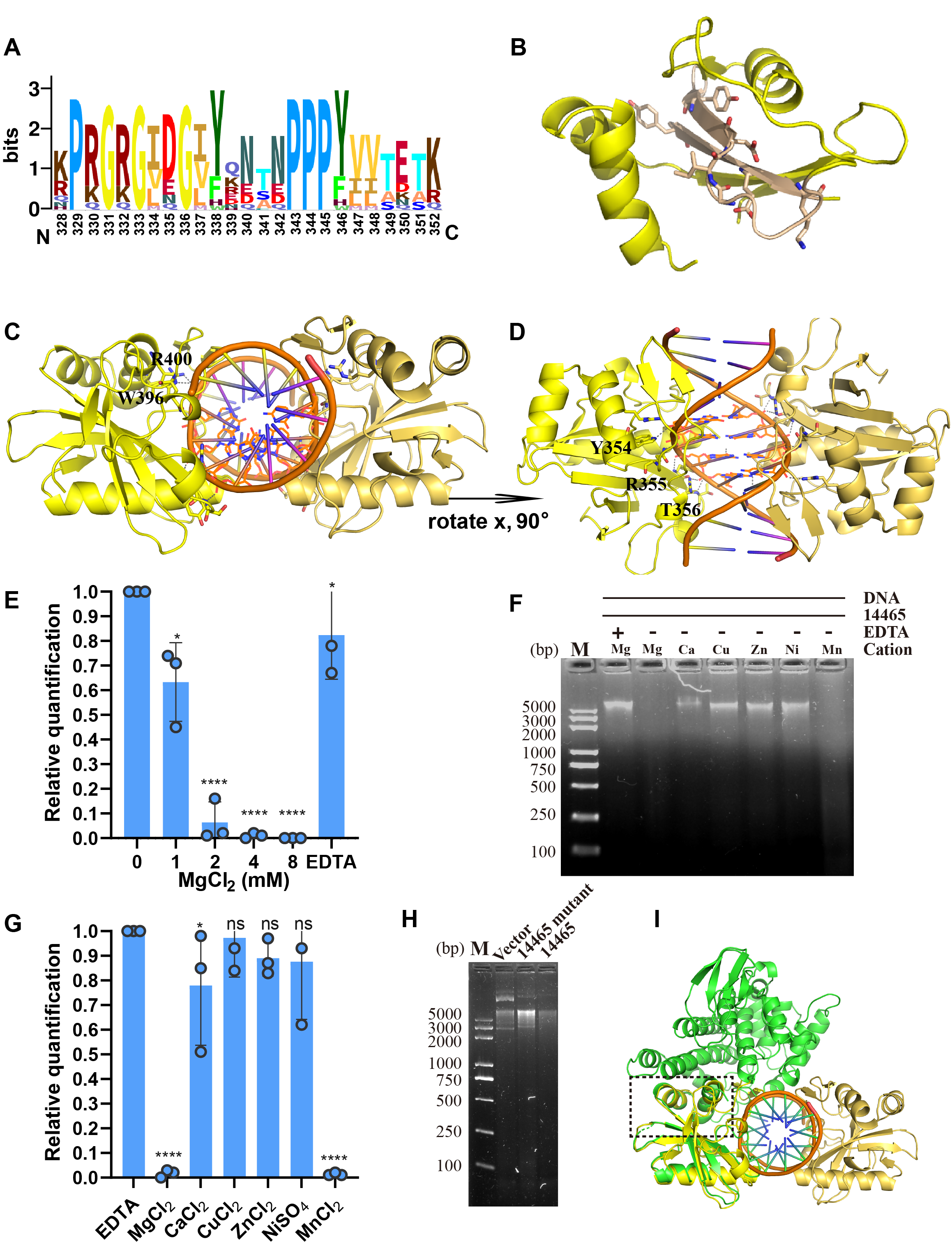
**

**Figure S4. Molecular docking of VP14465C with DNA.** (A) Multiple sequence alignment of Kx₃GExₙGIDx₂YxₙYx₃ExK motif in the homologs in NCBI by WebLogo. (B) The motif resides at the core of the three β-sheets in the structure of VP14465C (colored in wheet, the conseved residues are shown in sticks). (C, D) The details of the interaction between VP14465C and DNA docked by AlphaFold3. The residues involved are shown as sticks. (E) Relative quantitation of nuclease activity of VP14465 at different MgCl_2_ concentrations (0, 1, 2, 4, 8 mM). (F) Nuclease activity of VP14465 at different divalent cations. Plasmid DNA (pET28a) was used as the substrate, and 8 mM of MgCl_2_, CaCl_2_, CuCl_2_, ZnCl_2_, NiSO_4_, and MnCl_2_ were added respectively. All reactions were conducted at 37 °C for 30 min. Experiments were performed in triplicate, yielding consistent results, with representative data shown. (G) Relative quantitation of (F). (H) Agarose gel electrophoresis of genomic DNA extracted from *E. coli* expressing VP14465 or the mutant. (I) Superposition of VP14465C-DNA complex and VP14460-VP14465C complex, VP14465C-DNA complex is colored in yellow, VP14460-VP14465C complex is colored in green. The the overlapping region is marked with a rectangular dashed box.

.

**
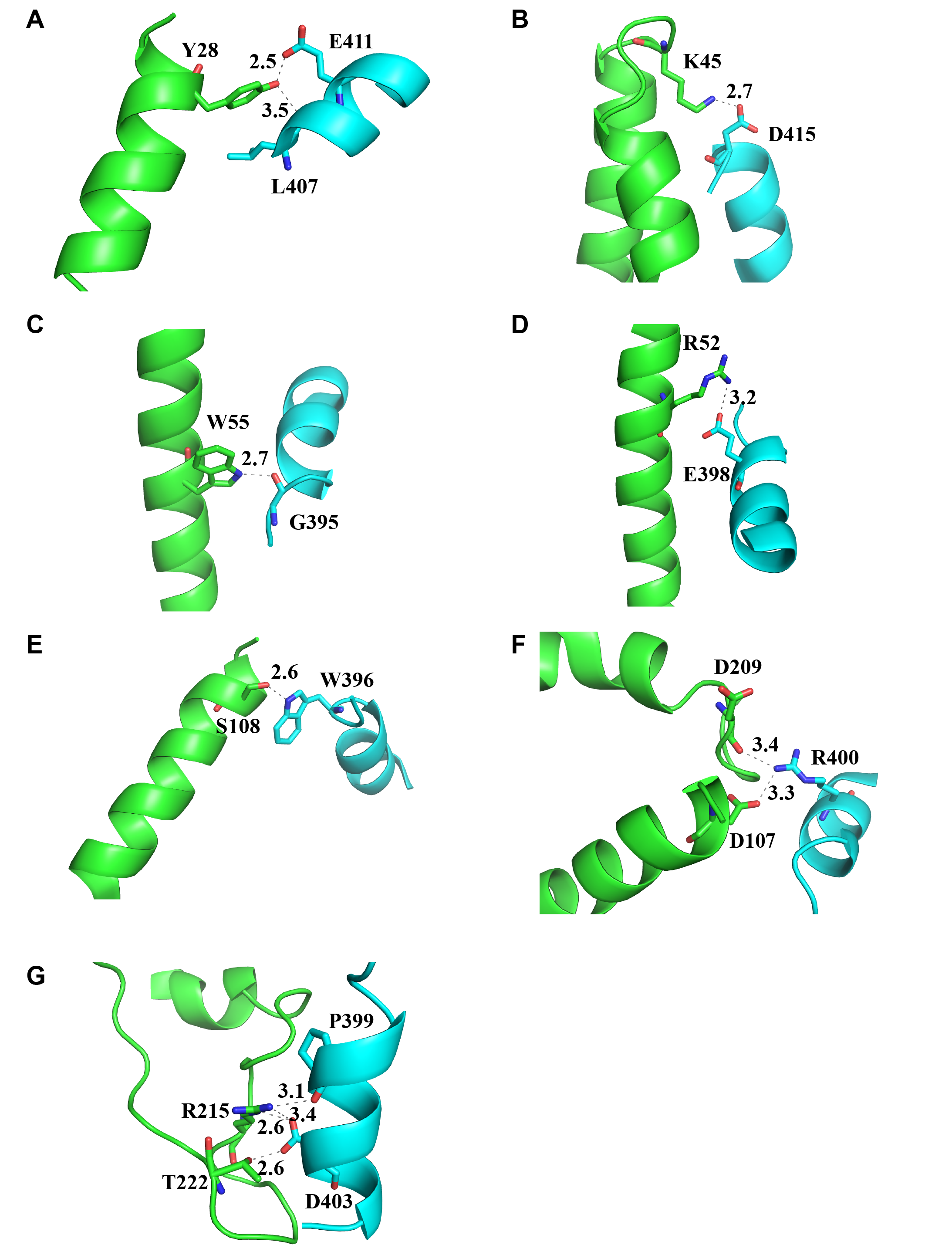
**

**Figure S5. Detailed view of the VP14460-VP14465 interaction interface.** (A-F) Close-up views of the intermolecular interactions between VP14460 (green) and VP14465 (cyan) at the binding interface. Key residues involved in hydrogen bonding and hydrophobic interactions are shown as sticks. Hydrogen bonds are indicated by grey dashed lines, with distances labeled in angstroms (Å). Residues mutated in this study (Y28, K45, R52, W55, D107, S108, D209 and T222 on VP14460, G395, W396, E398, P399, R400, D403, L407, E411 and D415 on VP14465) are highlighted.

**
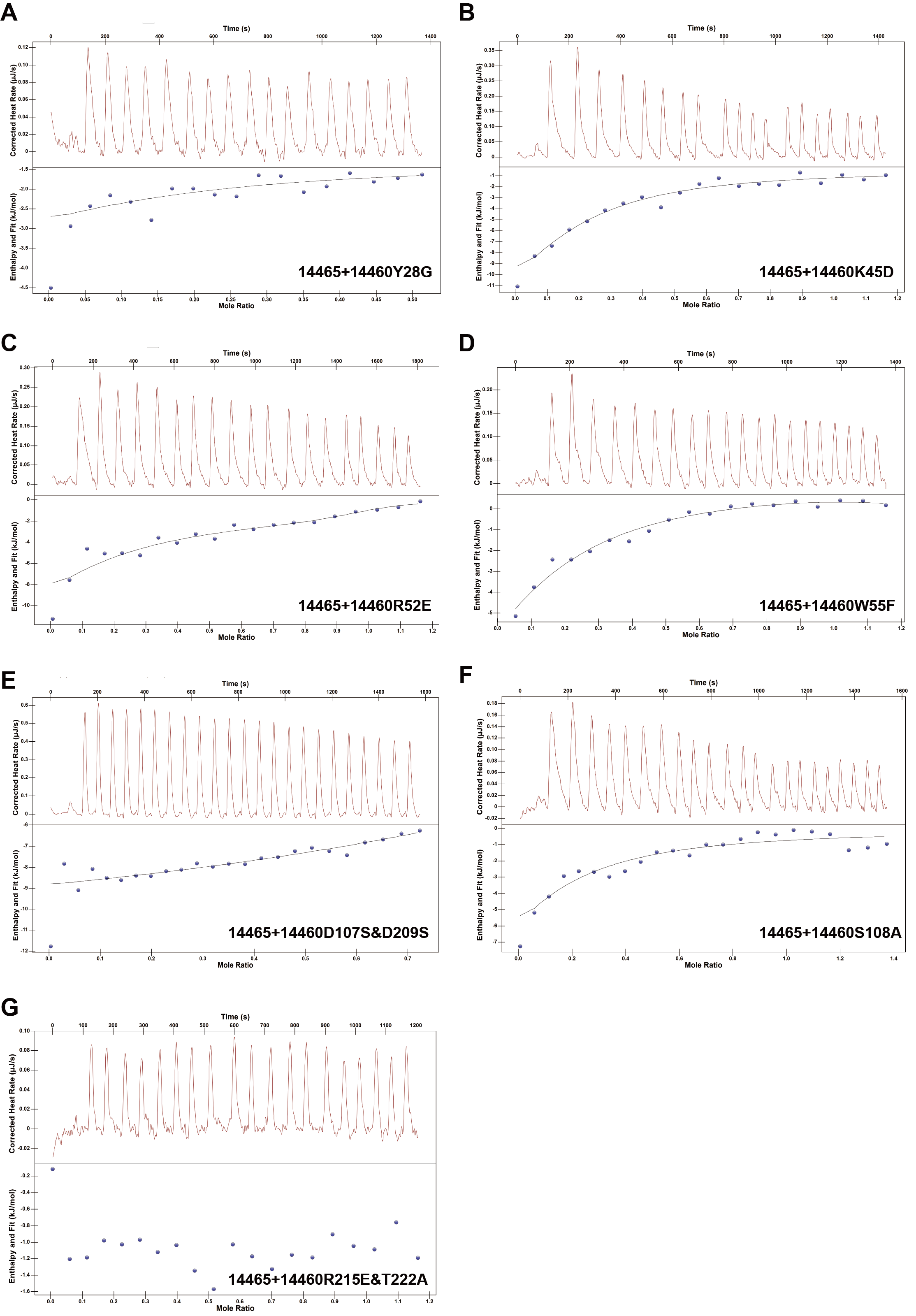
**

**Figure S6. Isothermal titration calorimetry of VP14465 peptide and VP14460 variants interaction.** ITC assays of VP14465 peptide and VP14460 variants Y28G (A), K45D (B), R52E (C), W55F(D), D107S&D209S (E), S108A (F) and R215E&T222A (G) at 25°C. The bottom graph illustrates the integrated heat for each injection of VP14465 peptide together with a fit, whereas the y axis represents the heat released per mole for each injection.


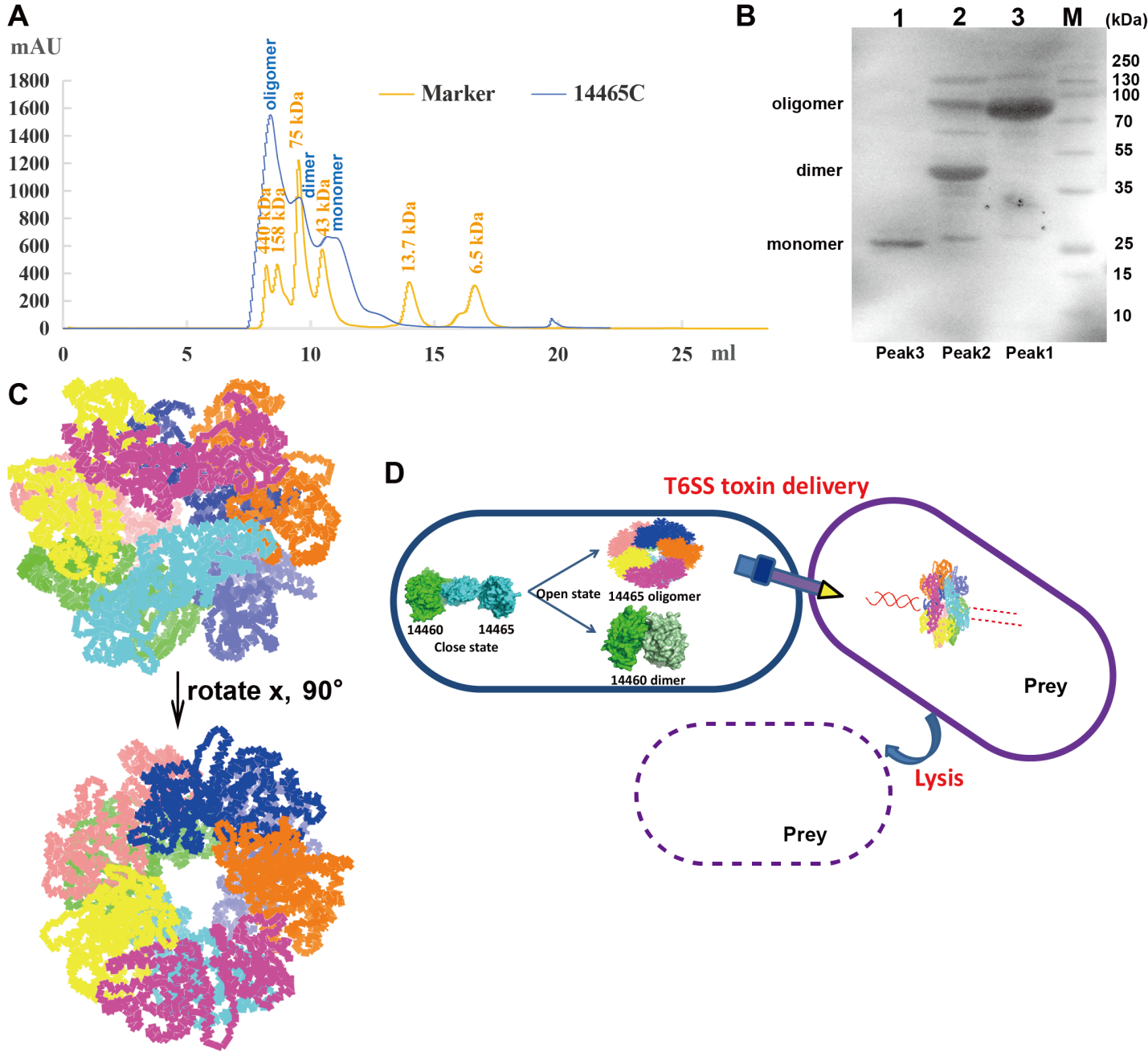


**Figure S7. A model for conformational changes of VP14460-VP14465 E-I module in bacterial competition.** (A) Superdex 75 elution profile/peaks of VP14465 monitored by absorbance at 280 nm. (B) Western blot bands formed by the three peaks of VP14465 sample. (C) The octameric structure of VP14465 predicted by AlphaFold3, each protein molecule is represented by a color. The two representations are rotated by 90 degrees. (D) Electrostatic surface potential of VP14465 complex with nucleic acid. Negative, positive, and neutral potentials are colored red, blue, and white, respectively. The nucleic acid is colored in yellow. (E) A model for the action mechanism of the VP14460-VP14465 E-I system delivered via the T6SS during bacterial competition. The structures resolved in this study are depicted in surface, and the predicted structures are depicted in ribbons.
